# Conference Reports: HYPERTEXT STANDARDIZATION WORKSHOP, Gaithersburg, MD, January 16–18, 1990

**DOI:** 10.6028/jres.095.031

**Published:** 1990

**Authors:** Jean Baronas

**Affiliations:** National Computer Systems Laboratory, National Institute of Standards and Technology, Gaithersburg, MD 20899

## 1. Introduction

Hypertext systems and technology are being developed and used by numerous industrial, academic and Government organizations today. “A Hypertext is a network of information nodes connected by means of relational links. A Hypertext System is a configuration of hardware and software that presents a Hypertext to users and allows them to manage and access the information that it contains,” as stated in reference [1]. The nodes in these systems may contain text, graphics, audio, and video, as well as source code or other forms of data. The nodes are meant to be viewed through an interactive browser and manipulated through a structure editor [2].

The members of the National Institute of Standards and Technology (NIST) National Computer Systems Laboratory (NCSL) Hypertext Competence Project (initiated in October 1988) held a Hypertext Standardization Workshop at NIST, on January 16–18, 1990, to provide opportunities for Hypertext system designers and users to discuss directions, timing, and requirements for Hypertext standardization. The Workshop was attended by 65 representatives from Hypertext system development and user communities. Workshop participants made progress toward generating a plan for Hypertext standards development which includes reference models, information interchange between different systems, and user requirements.

The Workshop program committee, comprised of members of the NIST Hypertext Competence Project, published a “Call for Papers” and then selected papers to be presented in the Workshop plenary sessions from responses to the “Call.” The papers that were presented in the Workshop included approaches to solutions for Hypertext modelling and standards, such as “The Trellis Hypertext Reference Model” by Richard Furuta and David Stotts of the University of Maryland at College Park, MD, “The Dexter Hypertext Reference Model” by Frank Halasz of the Xerox Palo Alto Research Center, Palo Alto, CA, and Mayer Schwartz of Tektronix Laboratories, Beaverton, OR, and “A Formal Model of Hypertext” by Danny Lange of the Technical University of Denmark. A total of 14 papers were presented during plenary sessions at the Workshop which covered aspects of the following:

### Workshop Paper Topic Overview

1. Descriptions of Hypertext systems using reference models,
2. Approaches to interchanging Hypertext documents and databases between heterogeneous systems, and
3. Applications of already-existing document processing standards within the Hypertext domain.

The presentations made during the Workshop plenary sessions provided the basis for interesting discussions in break-out sessions that were nm in parallel and followed the plenary sessions. The participants formed the Hypertext Models Discussion Group, Data Interchange Discussion Group, and the User Requirements Discussion Group for the break-out sessions.

## 2. The Hypertext Models Discussion Group

The Hypertext Models Discussion Group members defined a reference model as a “description of some domain that can be used to compare existing implementations in that domain, design new implementations, map out possible areas for standardization and show their relation to one another,” see reference [3]. The Group wrote a development plan for standardization, analyzed the differences and similarities of the before-mentioned Trellis, Dexter and Lange models, and organized the following taxonomy of Hypertext concepts.

### Taxonomy of Hypertext Concepts

1. Entities (objects) that a H3rpertext system must manipulate,
2. Properties of entities or of the entire Hypertext system,
3. Functions or operations including knowledge modification, navigation, system’s tailoring, interfaces, versioning, access control, and
4. Abstractions including schema, type, class, object, data models, encapsulation, layer.

The Group began ranking the concepts by their importance to Hypertext systems, took inventory of existing systems, and constructed an implementation model which is comprised of layers that represent system characteristics that are essential to Hypertext researchers and standards developers and those that should be covered by standardization efforts that are ongoing in other disciplines.

The Hypertext Models Discussion Group also developed a reference model to map out the areas where standards are needed. It represents a ranking of the most popular concepts in Hypertext systems, i.e., how central each is to Hypertext, to focus standardizing on those concepts that have the most widespread use. To select the area for standards development, the group analyzed the intersection of Hypertext features with the most widespread use and those that would be best standardized by members of other research communities, such as the computer human interface, database, object oriented programming, and windowing domains.

## 3. The Hypertext Data Interchange Discussion Group

The Hypertext Data Interchange Group discussions focused on making a distinction between delivery and archival interchange concepts. The Group summarized that a delivery interchange standard could be one that is usable by a conforming Hypertext system, without translation. In the near term, such a standard would be difficult to achieve due to the different data storage and indexing approaches that exist in various commercially- available Hypertext systems, in addition to the different methods of data representation and presentation that are used.

The development of an archival interchange standard was discussed. In this approach, the user would store Hypertext in a vendor-specific format at both the source and target systems, with a translation to and from the “archival interchange” format. Changes or updates would be stored in the archival store before reaching the other platforms. The Group suggests that the development of the “archival interchange” approach could be done in the nearer term, as compared with the “delivery interchange” approach.

The Group discussed the interchange formats that were covered by Workshop contributions and noted that all adopted the concept of partitioning interchange into data objects, Hypertext anchors, and links. The contributions were written with different terminology; however, there was a fundamental conformance to the layering approaches that they represented. According to reference [4] “Most of the proposals included the Standard Generalized Markup Language (SGML) or SGML-like systems as a basis for tagging text. It was agreed that SGML was a reasonable basis for further interchange experiments between existing Hypertext systems.” The Hypertext Data Interchange Group also identified other Hypertext characteristics that are fundamental to interchange and suggested that they be discussed in future standards sessions.

## 4. The Hypertext User Requirements Group

The Hypertext User Requirements Group overviewed Hypertext applications and identified factors that account for different Hypertext design concepts (enabling technology, document standards initiatives, market pressure, and increased academic interest). The Group discussed two different views of what it termed the Hypertext Design Space: a dimensional view and an edit or not to edit view. The dimensional view includes the user dimension (single users vs. groups, vs. multiple users that are unrelated), the information dimension (creation vs. conversion), the task dimension (specific vs. general), and the interface dimension (static vs. dynamic). The edit or not to edit view covers a framework for understanding Hypertext Design Space that partitions applications by whether or not users can edit the content of Hypertext units, and the links between them, from those that are read-only, i.e., the user can only browse (read) the units and the links cannot be changed.

The Group concluded that users and manufacturers would benefit from the development of shared specifications for Hypertext functions that are carried out by a Hypertext user interface on entities that are managed by a Hypertext storage layer. Such specifications would be useful if they had the following characteristics:

### Hypertext User Specifications Characteristics

1. Fit in proposed Hypertext Reference Model,
2. Maintain independence from presentation specifications, and
3. Define operational semantics of the system.

The Group maintained that if these specification goals are satisfied, standards for Hypertext functions can be organized into subsets that fit into different parts of the Hypertext Design Space. An initial list of functions that can be used to define the interoperability of Hypertext systems within a given Hypertext Design Space includes those that effect a Hypertext unit, link, or composite unit follows:

### Initial List of User Functions

1. Create,
2. Edit,
3. Delete,
4. Publish,
5. Indicate current unit,
6. Move to another unit,
7. Indicate presence of an expandable structure.

Another phase of the Group discussions included the importance of developing definitions and rules for various user functions. The User Requirements Group concluded their work by listing research agenda that are needed to help define semantics for Hypertext functions and new measures for Hypertext that describe characteristics which are relevant to user performance. These agenda include the following.

### Hypertext Research Agenda

1. Evaluating hypertextability of a document or a document collection,
2. Validating Hypertext conversion which includes methods and tools for measuring the amount of Hypertext that has been successfully converted,
3. Measuring Hypertext readability, and
4. Analyzing the intellectual property issues of Hypertext.

## 5. Recommended Directions for Hypertext Standards

In the final plenary session, the Workshop participants discussed the possibilities of establishing a more formal Hypertext study group within accredited standards bodies. Major conclusions made in the final plenary discussions can be summarized as the following.

### Workshop Conclusions

1. The discussion groups should continue efforts,
2. NIST should sponsor a follow-up workshop,
3. Decision should be made as to the development of a standards committee with official status in American National Standards Institute (ANSI).

## 6. Workshop Proceedings

More detailed reports from the discussion groups, in addition to the technical papers that were presented during the Workshop plenary sessions are published in NIST Special Publication Number 500-178, Proceedings of the Hypertext Standardization Workshop; January 16–18, 1990; National Institute of Standards and Technology, edited by Judi Moline et al. March 1990, which is available from the United States Government Printing Office, Superintendent of Documents, Washington, DC 20042.

## 7. NIST Hypertext Systems and Applications Workshop, October 1989

Preliminary to the Workshop, the Competence Project sponsored a Hypertext Systems and Applications Workshop at NIST on October 23, 1989, which consisted of overviews of Hypertext systems and applications, in addition to discussion of the need for a Hypertext reference model and the pursuit of Hypertext standardization. Hypertext system vendors provided system demonstrations for the October 1989 Workshop participants. The Hypertext Standardization Workshop was held in response to the request for the beginning of discussions of Hypertext standardization that prevailed in the October Workshop. Both events shared NIST Hypertext Competence Project research with NIST staff members and built close relationships between industry and Competence Project personnel. For further information, please contact Jean Baronas, NIST, Technology Building, Room B263, Gaithersburg, MD 20899, telephone: 301-975-3338, electronic mail: baronas@asl.ncsl.mst.gov.

The author, Jean Baronas, is a technical staff member in the NCSL and is also a member of the NIST Hypertext Competence Project. Other project members are Len Gallagher (Project Leader), Judi Moline, and Dan Benini of the NCSL.
